# Operative technique and morbidity of superficial femoral vein harvest

**DOI:** 10.1007/s00772-016-0170-6

**Published:** 2016-08-01

**Authors:** A. Neufang, S. Savvidis

**Affiliations:** Dr. Horst Schmidt Klinik Wiesbaden, Ludwig Erhard Straße 100, 65199 Wiesbaden, Germany

**Keywords:** Prosthesis infection, Autologous vein, Superficial femoral vein, Operative procedures, Complications, Protheseninfektion, Autologe Vene, Vena femoralis superficialis, Operationstechnik, Komplikationen

## Abstract

**Background:**

The use of autologous superficial femoral veins (SFV) as an arterial or venous substitute represents a valuable technique in modern vascular surgery with versatile indications. The SFV autografts exhibit excellent control of infection and durable long-term results in terms of graft patency in prosthetic or arterial infections. In cases of elective use of the SFV, duplex ultrasound evaluation of the deep leg vein system should be implemented to confirm the patency of the profunda femoris vein.

**Material and methods:**

The SFV can be harvested distal to the adductor hiatus with a proximal portion of the popliteal vein but should not exceed the level of the knee joint. Formation of a stump of the proximal SFV must be avoided. Simultaneous harvesting of the ipsilateral greater saphenous vein should be avoided to reduce the risk of significant chronic edema.

**Results:**

Early postoperative swelling of the donor leg can be expected but resolves spontaneously in most cases. Chronic mild edema of the leg with a possible indication for compression therapy may occur in up to 20 % of cases but severe complications are very rare if the anatomical borders for vein harvesting are respected. Temporary therapeutic anticoagulation after vein harvest is subject to individual decision making.

**Conclusion:**

Duplex ultrasound is a reliable tool to assess the residual deep and superficial venous system in the long term. Excellent graft function and the tolerable adverse effects of vein harvest on the donor leg justify the use of the SFV in arterial and venous vascular surgery if indicated.

## Zusammenfassung

**Hintergrund:**

Die Verwendung der autologen V. femoralis superficialis (VFS) als arterieller oder venöser Gefäßersatz stellt ein wertvolles Instrument in der modernen Gefäßchirurgie mit vielfältigen Indikationsmöglichkeiten dar. Insbesondere in der septischen Gefäßchirurgie führt die Verwendung der VFS zu ausgezeichneten Resultaten in Hinsicht auf die Beherrschung der Infektion und die langfristigen Graftfunktion.

**Materialien und Methoden:**

Bei elektiver Indikationsstellung zur Verwendung der VFS sollte eine duplexsonographische Evaluation des tiefen Beinvenensystems erfolgen und speziell die Offenheit der V. profunda femoris überprüft werden. Die VFS kann auch über den Adduktorenschlitz hinaus mit einem proximalen Anteil der V. poplitea entnommen werden. Die Entnahme sollte dabei das Niveau des Kniegelenks nicht überschreiten. Eine Stumpfbildung der proximalen VFS ist immer zu vermeiden. Die simultane Entnahme der gleichseitigen V. saphena magna soll zur Vorbeugung einer persistierenden chronischen Schwellneigung nicht erfolgen.

**Ergebnisse:**

Eine vorübergehende frühpostoperative Schwellneigung des Beines bedarf in der Regel keiner spezifischen Therapie. In bis zu 20 % der Fälle kann als Folge der Entnahme ein anhaltendes mäßiges Ödem mit Indikation zur Kompressionstherapie entstehen. Gravierende Komplikationen am tiefen Venensystem sind aber bei Einhaltung der anatomischen Begrenzungen im Rahmen der Entnahme nicht zu erwarten. Eine temporäre therapeutische Antikoagulation nach Venenentnahme muss individuell entschieden werden.

**Schlussfolgerung:**

Das residuelle tiefe und oberflächliche Venensystem kann im Langzeitverlauf verlässlich mittels Duplexsonographie beurteilt werden. Die exzellente Graftfunktion und die angesichts der langfristigen Ergebnisse eher seltenen und tolerierbaren negativen Auswirkungen auf den venösen Rückstrom rechtfertigen den Einsatz der VFS in der arteriellen und venösen Gefäßchirurgie bei sorgfältiger Indikationsstellung.

## Introduction

It is thanks to Martin Schulman that the significance of deep leg veins, the superficial femoral vein (SFV), as an autologous vascular graft in arterial and venous vascular surgery was recognized and that it is finding increasingly broader applications. He used the SFV as an autologous graft in peripheral bypass surgery for the first time in 1974 and reported comparable results using the SFV and the great saphenous vein (GSV) as femoropopliteal bypass material in 1987 [1–3]. Although the results of surgery performed largely for critical ischemia due to artherosclerotic occlusion of the femoropopliteal axis were good with secondary patency of up to 83 % at 4 years, Schulman was subjected to massive criticism due to the significantly more invasive SFV harvesting technique compared with conventional GSV harvesting. Severe complications due to restricted venous outflow following SFV harvest were feared and the method was criticized for its experimental nature [4]; however, Schulman et al. reported early on only slightly increased leg edema following SFV harvesting compared with GSV dissection and harvesting [1]. Schanzer et al. confirmed this finding as early as 1991 in another investigation, which also found only mild calf enlargement in the affected leg following SFV harvest and supported the role of the SFV as a replacement material for large veins or as an arterial graft [5]. Due in particular to the efforts of Clagett et al. in the USA and Nevelsteen et al. in Europe, the use of the SFV quickly met with acceptance and widespread application as a technique for autologous vascular reconstruction in septic vascular surgery for graft infection in the aortoiliac region or in the case of primary arterial infection [6, 7].

## Indications for use of the superficial femoral vein

The SFV is now well established as an autologous graft in reconstructive arterial and venous vascular surgery. Histomorphological investigations have shown that the collagen and elastin composition of the vein wall and its associated compliance is comparatively similar to that of an autologous artery, thereby also explaining the low tendency toward marked myointimal hyperplasia [8]. In addition to the excellent data on fully autologous reconstruction in septic vascular surgery involving the repair of infected conventional vascular grafts or mycotic aneurysms [6, 7, 9–16], results on the successful use of this approach in infected stents or stent grafts are also available [17–19]. This also applies to the thoracic or thoracoabdominal aorta in individual cases [20, 21]; however, the SFV has also proved its worth as a permanent conduit for other indications. In the case of failed conventional or endovascular repair it can be effectively deployed in aortoiliac reconstruction due to arterial occlusive disease in the pelvic region [22, 23]. It can be successfully used as peripheral bypass material [1–3, 24–27] as well as in the reconstruction of arterial and venous visceral [28, 29] and supra-aortic arterial vessels [30, 31]. There are indications for its use in the reconstruction of large veins [32], such as the superior [33–38] and inferior [39–41] vena cava, as well as in cancer surgery [42, 43]. A great deal of experience has already been gained with the SFV in the creation of arteriovenous fistulas for hemodialysis access [44–46] (see Table 1 for a summary of possible indications and representative results).

**Table 1:** Indications for use of the superficial femoral vein

Indication	Author	Year	Localization	Number of procedures	Patency/particular features
Graft infection/arterial infection in abdominal aortic and iliac vessels	Clagett et al. [6]	1993	Aortoiliac	20	100 %
	Nevelsteen et al.[16]	1995	Aortoiliac	15	13/15 (all survivors)
	Clagett et al. [10]	1997	Aortoiliofemoral	41	100 % 5 years secondary
	Franke and Voit[14]	1997	Aortoiliac	7	100 %
	Daenens et al. [11]	2003	Aortoiliac	49	91 % 5 years primary
	Ehsan and Gibbons [13]	2009	Aortoiliac	46	91 % 5 years secondary
	Ali et al. [9]	2009	Aortoiliac	187	91 % 6 years secondary
	Dorweiler et al. [12]	2014	Aortoiliac	86	97 % 5 years secondary iliac
	Heinola et al. [15]	2015	Aortoiliofemoral	55	80 % Intervention-free 6 years
EVAR stent graft infection	Fatima et al. [18]	2013	Aortoiliac	2	–
	Davila et al. [17]	2015	Aortoiliac	4	–
Iliac stent infection	Sternbergh and Money [19]	2005	Common iliac artery	Case report	5 year patency and survival
Thoracic and thoracoabdominal aorta	Tambyraja et al. [21]	2003	Thoracoabdominal aortic patch graft infection	Case report	Survival and resolution
	Okamoto et al. [20]	2012	Descending aorta graft infection	Case report	Survival and resolution
Aortoiliac occlusive disease^a^	D’Addio et al. [22]	2005	Crossover bypass	54	90 % 5 years secondary
Premature atherosclerosis	Jackson et al. [23]	2004	Aortofemoral bypass	31	100 % 5 years
Peripheral bypass material	Schulman et al. [1]	1987	Femoropopliteal bypass	76	83 % 5 years secondary
	Sladen et al. [26]	1994	Infrainguinal bypass	25	80 % 2 years secondary
	Wozniak et al. [27]	1998	Infrainguinal bypass	32 (PTFE composite)	56 % 4 years secondary
	Gibbons et al. [24]	2003	Infrainguinal bypass	12	76 % 4 years secondary
	Kaczynski and Gibbons [25]	2011	Infrainguinal bypass	20	78 % 12 months
Visceral arterial vessel reconstruction	Modrall et al. [28]	2003	Visceral artery bypass/replacement	20	100 % 2 years
Supra-aortic arteries	Modrall et al. [30]	2002	Supra-aortic bypass (subclavian artery, carotid artery, axillary artery)	18	100 % 4 years assisted
	Schindler et al. [31]	2002	Subclavian artery replacement for mycotic aneurysm	Case report	Vein rupture in persistent infection!
Large vein replacement	Hagino et al. [32]	1997	Vena cava and peripheral veins	7	100 % 2 years
	Kanno et al. [36] Gladstone et al. Klima et al. [38] Erbella et al. [33] Eshtaya et al. [34] Kennedy and Palit [37]	1981 1985 1994 [35] 2006 2008 2010	Superior vena cava	Case report or series	–
	Schwartz et al. [41] Bower et al. [39] DuBay et al. [40]	1991 2000 2009	Inferior vena cava	Case report or series	–
Injury to the superior mesenteric vein	Tulip et al. [29]	2012	Superior mesenteric vein replacement	Case report	1 year
Cancer surgery	White et al. [43]	2005	Iliac vein sarcoma	Case report	–
	Lee et al. [42]	2010	Portal vein system	15	–
Arteriovenous fistula	Gradman et al. [46]	2001	AV shunt	25	86 % 12 months secondary
	Gilbert and Gibbs [45]	2011	AV loop	16	90 % 12 months secondary
	Bourquelot et al. [44]	2012	Dialysis shunt	70	56 % 9 years

*AV* arteriovenous, *EVAR* endovascular aortic repair, *PTFE* polytetrafluoroethylene ^a^femorofemoral bypasses were placed for aortoiliac disease

## Anatomy of the deep venous system

The deep venous system of the leg is made up of the structurally double lower leg veins, the popliteal vein, the SFV, the deep femoral vein and the common femoral vein. The veins of the calf, the so called tibial and peroneal veins are doubled and form after their union the popliteal vein in the upper calf. The popliteal vein follows a spiral-shaped course around the popliteal artery and lies in the proximal popliteal fossa dorsolateral to the artery. Once it has passed through the adductor hiatus, the popliteal vein proximally becomes the SFV, which lies posterolateral to the accompanying artery in the adductor canal, along the course of which it receives numerous tributaries. The vein itself is crossed by arterial tributaries of the accompanying superficial femoral artery, which, in the case of existing femoral peripheral arterial disease (PAD) particularly in the distal portion, can represent important collaterals. The SFV and the deep femoral vein join to form the common femoral vein 4–12 cm below the inguinal ligament. The deep femoral vein lies anterior to the deep femoral artery. Worthy of note is the lateral femoral circumflex vein, an important branch of the deep femoral vein extending laterally, which is susceptible to injury during dissection in this region [47].

## Preliminary diagnostic work-up in planned superficial femoral vein harvest

The decision to use the SFV for arterial or venous reconstruction may be taken either in the acute setting during surgery or earlier on at the preliminary stage of planning elective surgery. The status of venous collateral circulation of the popliteal vein via the deep femoral vein in a central direction, which will be needed in the future, is the key to the planned harvest of the SFV. The requisite central venous outflow in the common femoral vein is only guaranteed long-term if the deep femoral vein is intact. This phenomenon is already known from venous pathology of the thigh as axial transformation, although the author of that particular study [48] described this structural variation in relation to postthrombotic disease or obliteration of the SFV. Raju et al. described the deep femoral vein as the dominant or only venous outflow vessel in the diseased leg in up to 45 % of extremities with venous stasis. The deep femoral vein is also able to accommodate increased circulation due to a corresponding increase in caliber [48].

Information on the extent of possible previous arterial surgery in the groin and on the affected leg is crucial in cases where use of the SFV is planned. It is essential to establish, possibly from old surgical reports, whether the deep femoral vein has been affected by previous dissection of the deep femoral artery or profundaplasty or possibly dissected in a targeted manner in the course of artery exposure. Where this is the case, harvesting from a leg that has undergone surgery of this kind is naturally prohibited. Previous harvest of the GSV, on the other hand, does not represent a contraindication, assuming future outflow is guaranteed by the deep femoral vein [49].

**Fig. 1:**
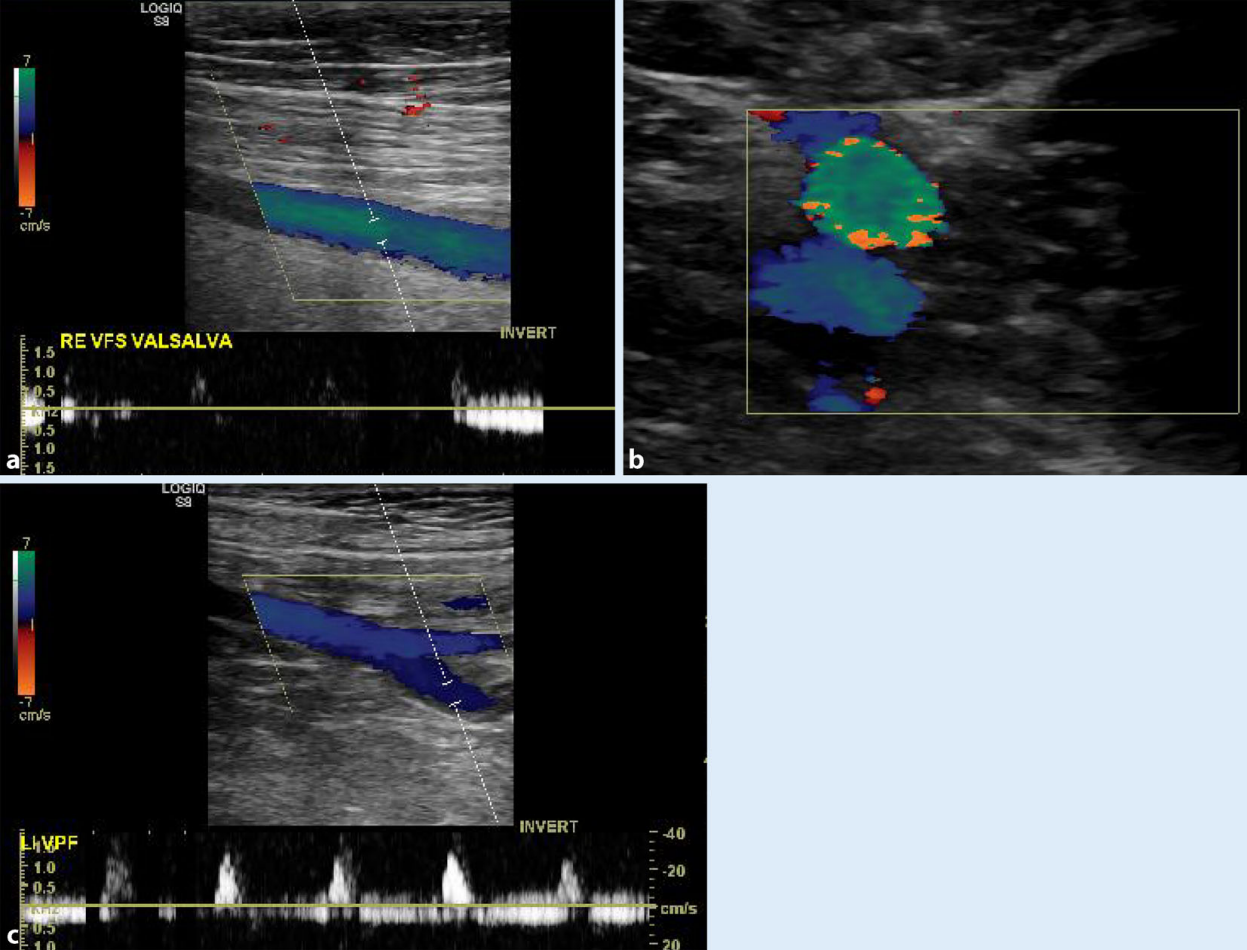
Duplex sonography showing **a** a normal and **b** a bifid superficial femoral vein and **c** junction of the deep femoral vein

Schulman et al. performed SFV harvesting only after prior phlebography of the deep venous system and upon confirmation of an intact deep femoral vein [2, 3]. This approach, however, has largely disappeared from the clinical routine and is no longer a routine diagnostic measure due to the broad availability of duplex ultrasound. With the exception of emergency situations, preoperative evaluation of the superficial and deep venous system should always be performed using duplex ultrasound. In the case of a spontaneous, intraoperative decision to use the SFV without preoperative duplex examination, the venous confluence between the SFV and the deep femoral vein in the proximal thigh should be surgically explored for integrity of the profunda femoris vein in a first step and only after this should further dissection of the SFV take place. If postthrombotic changes to the SFV are found the vein should not be used [47]. Duplication of the deep venous system in the thigh is known to be present in up to 25 % of individuals and can be easily identified using duplex sonography [50]; however, even a duplicated vein can be used as a graft, assuming it has the appropriate caliber (Fig. 1a,b). One usually sees a venous caliber of 5–9 mm on duplex sonography. If the use of the vein from both legs is anticipated in the context of extensive revision of an infected central aortic graft or an aortobifemoral graft bypass, preoperative evaluation of both legs for possible vein harvesting is essential. In such cases, this also applies to the status of the previously operated femoral artery. Duplex sonography is well-suited to confirming the patency of the profunda femoris vein (Fig. 1c), thereby simplifying planning in the case of extremities that, in some cases, have undergone multiple previous surgeries.

## Procedure for the dissection and harvest of superficial femoral veins

**Fig. 2:**
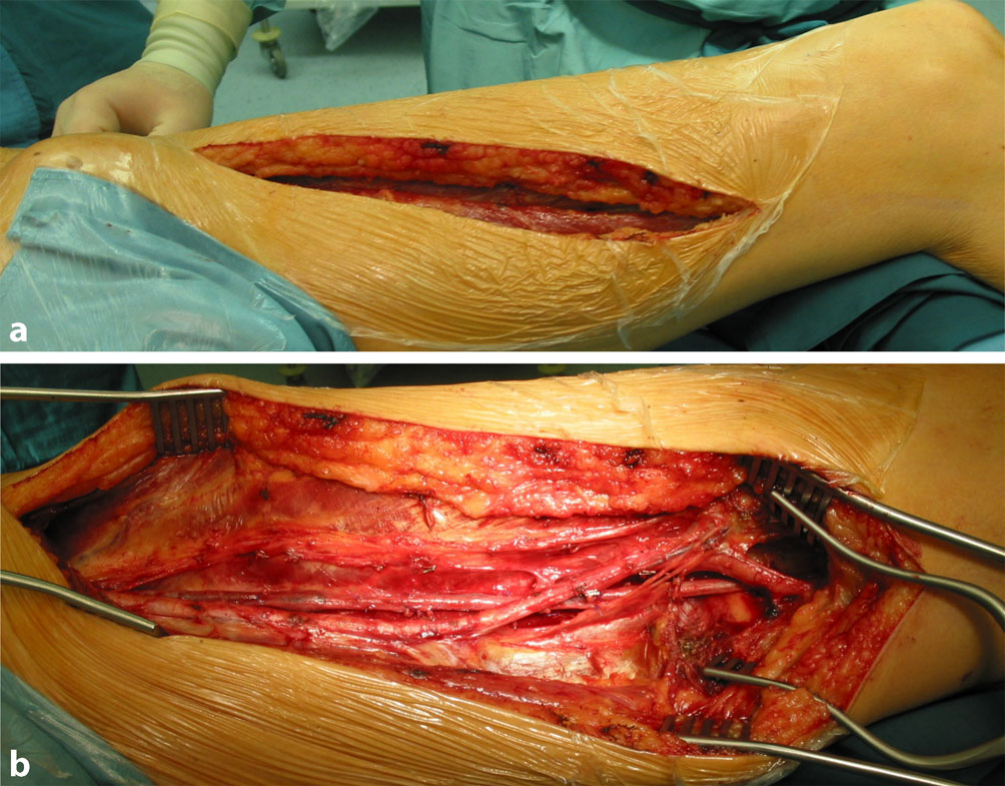
**a **Incision and **b** exposure for the dissection and harvesting of the superficial femoral vein

In principle, the possible use of the SFV in elective or emergency vascular surgical interventions should already be considered when positioning and draping the patient. The same also applies in elective cancer surgery where potential vascular involvement is anticipated and possible SFV harvesting has been included in the surgical concept following preoperative, interdisciplinary consultation. Both legs, including the groin region, should always be completely disinfected and draped. In the case of aortoiliac graft infection, draping should also take the creation of a cross-over bypass as a possible variant into consideration and leave the suprapubic region visibly exposed with a view to bypass routing. As in bypass surgery, it is advisable to place a cylindrical cushion under the knee, thereby facilitating dissection in the proximal popliteal fossa. With the leg slightly externally rotated and the knee flexed, a longitudinal incision is made in the thigh from a proximal direction along the ventral course of the sartorius muscle, possibly extending to the level of the knee where necessary (Fig. 2a). A long segment of the adductor canal is opened in front of the muscle and the SFV is exposed in a proximal and distal direction while preserving the small branches that perfuse the muscle with blood (Fig. 2b). The superficial femoral artery is also preserved or, in the case of pre-existing occlusion, meticulous attention is paid to the preservation of distal collaterals to the popliteal artery. The unnecessary sacrifice of collaterals of this kind can, in the worst case, cause severe ischemia in the affected leg.

The required length of the SFV is then completely harvested from its bed in a step by step approach. Early ligation of the vein is not beneficial; however, it is essential that blood flow in the vein is maintained throughout the entire dissection phase in order to avoid stagnation thrombosis in the SFV. It is sometimes necessary to carefully dissect around accompanying arterial structures without compromising these. As part of this process, the venous side branches are ligated as far as possible peripherally using (double) clips and, in the case of planned implantation in an infected site, the central stump of the venous branch is oversewn to the SFV with non-absorbable sutures (5.0 or 6.0 polypropylene) using a transfixion ligature. If implantation is performed in an uncontaminated area, (possibly double) conventional ligation using a non-absorbable suture (e. g. 4.0 Mersilene) can be carried out; however, a number of experienced authors have pointed out the risk of postoperative bleeding due to loosening of the ligature [51].

**Fig. 3:**
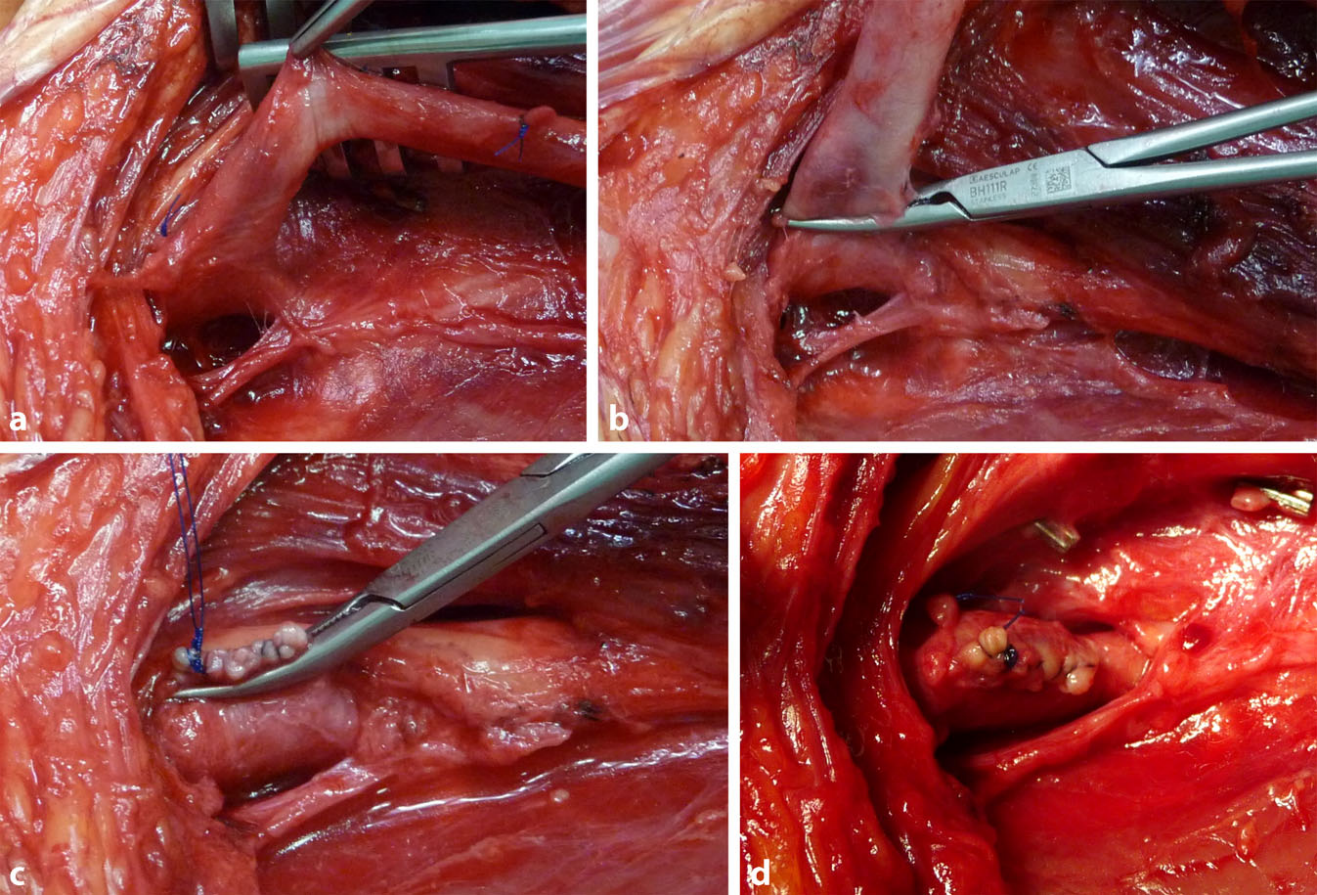
Dissection of the profunda vein and reconstruction of the common femoral vein junction: **a** exposure of the junction between the deep femoral vein and the common femoral vein, **b** the superficial femoral vein (SFV) is clamped tangentially above the profunda vein junction, **c** the superficial femoral vein is transsected and continuous oversewn with polypropylene sutures (5.0 or 6.0) and **d** vascular continuity is verified

**Fig. 4:**
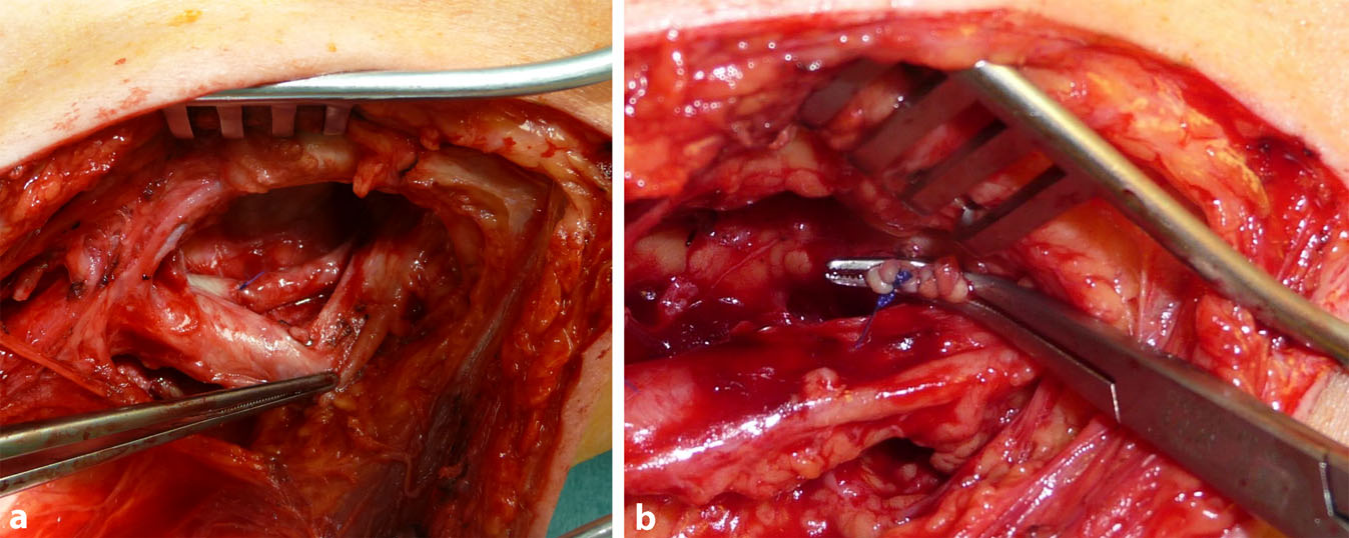
The popliteal vein is dissected and oversewn: **a** dissection of the popliteal vein is performed lateral to the popliteal artery and **b** continuous oversewing of the popliteal vein stump with polypropylene sutures (5.0 or 6.0)

**Fig. 5:**
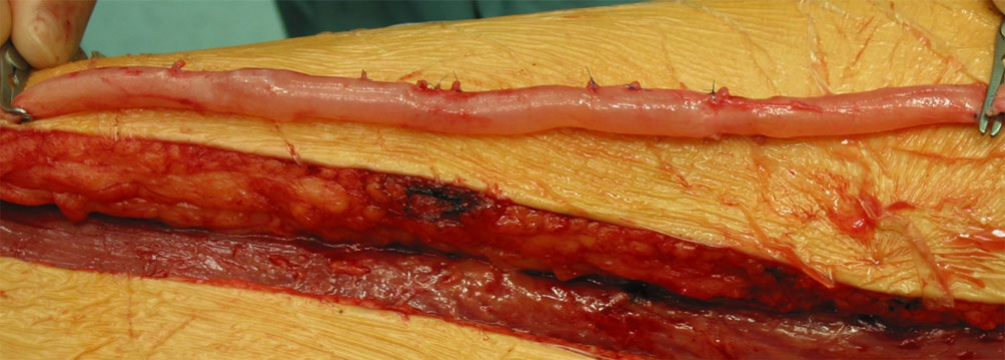
Harvested superficial femoral vein: side branches are oversewn with polypropylene sutures (5.0 or 6.0). To assess for leaks, the vein is filled with heparinized NaCl solution

Centrally, the junction of the deep femoral vein and the common femoral vein is visualized by exposing the venous confluence in such a way that it can be clamped tangentially and the SFV can be excised over the clamp using a scalpel or scissors. The excision margin is then sutured with a continuous polypropylene suture over the horizontal clamp in such a way that a harmonious junction between the deep femoral vein and the common femoral vein is created without constricting the profunda femoris vein (Fig. 3a–d). It is essential to avoid a proximal stump on the SFV due to the risk of possible thrombus formation and subsequent ascending phlebothrombosis [51]. In the knee joint, the distal vein stump is tied off peripherally according to the length required and following vein excision, oversewn with a continuous suture or ligated (Fig. 4a,b). Clamping and excision of the vein is only performed once all venous side branches have been identified and ligated. Prior to ligation, heparin can then be administered systemically where necessary, if arterial dissection has possibly already been completed by a second team and the arterial component of surgery can be initiated. The harvested vein is then removed from its bed without damaging arterial collaterals, carefully distended by means of filling with heparinized NaCl solution and inspected for possible leaks (Fig. 5). Where necessary, these are oversewn with a thin polypropylene suture.

**Fig. 6:**
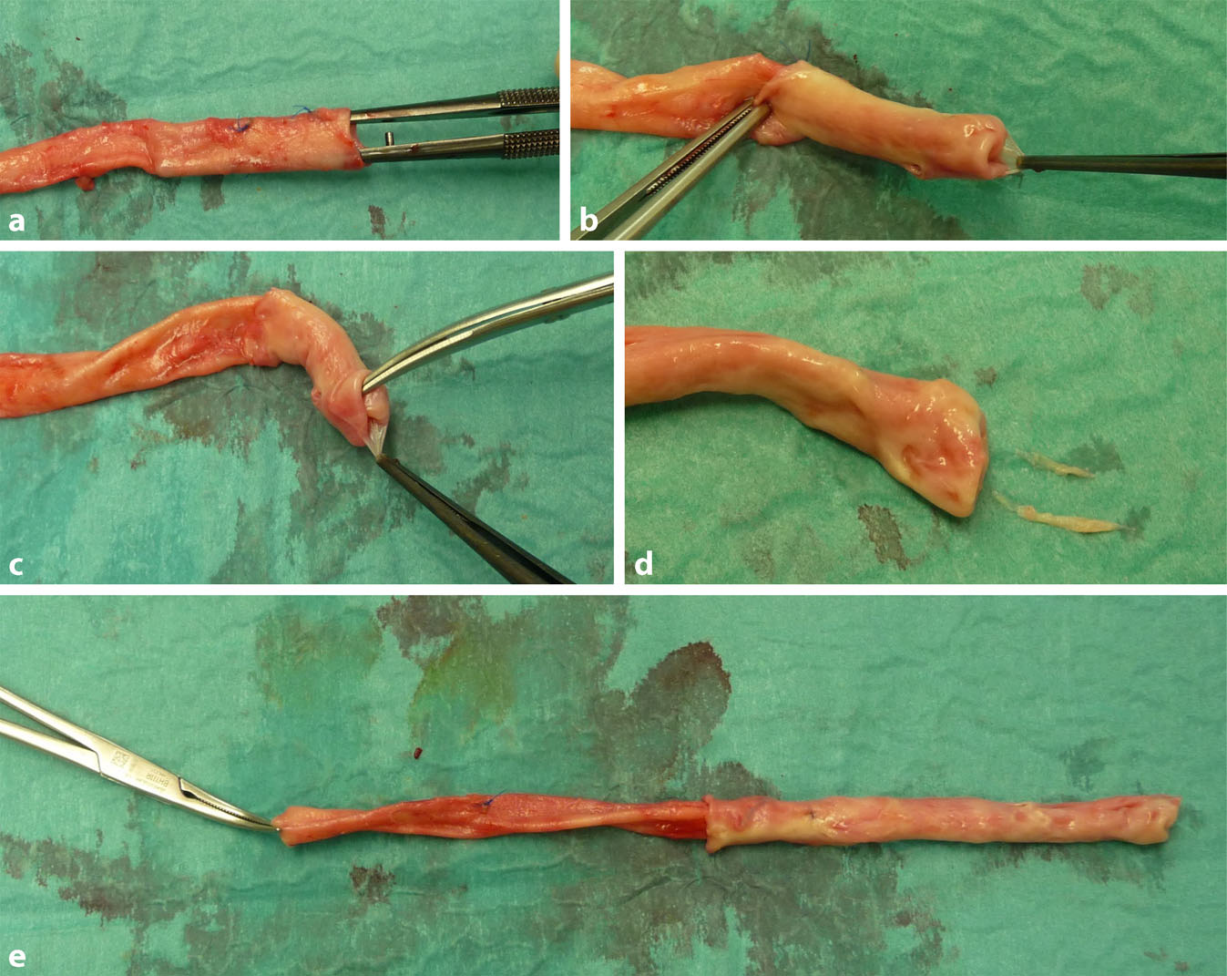
Eversion and excision of the venous valves: **a** the first valve is gripped with fine forceps introduced into the lumen, **b** manual traction is applied to the valve using forceps and the vein is everted in a distal direction, **c** the valvular leaflet is excised using fine scissors under direct vision, **d** everted vein with excised valves and **e** small forceps on the distal vein prevents complete eversion of the vein

The harvested vein can be grafted either in a reverse position (with preserved venous valves) or in a non-reversed position if valve destruction has taken place. The vein normally tapers peripherally, meaning that, as a basic principle, one should consider orthograde grafting and venous valve removal. The author Dr. Neufang always removes venous valves as a matter of routine, not least to prevent possible clot formation or subsequent stenosis on the comparatively large and rigid valves. Venous valves are destroyed according to the Valentine technique using a retrograde Mills valvulotome, as with the GSV, by cutting the valvular leaflet [26, 47]. As SFV valves can be relatively rigid, this type of valve destruction can be challenging in individual cases and may cause damage to the vein wall. For this reason, the author Dr. Neufang prefers the technique described for open valve excision via stepwise eversion of the vein [51]. Coming from a proximal direction with long, fine forceps, the first valve is packed and the vein is then everted distally until the valve plane is completely visible. The two leaflets are then sparingly excised under direct vision (magnifying spectacles) with fine scissors without damaging the wall; then, after gripping the next valve leaflet, the next valve plane is everted until stepwise all valves have been exposed and excised. At the same time, a fine clamp on the distal vein end prevents unintentional complete eversion of the vein (Fig. 6a–e). Finally, following complete valve excision, the vein is returned to its original direction. If an inspection of the vein by means of careful distension reveals marked ectatic segments (possibly in the area of the valve planes) with a diameter greater than 1 cm, continuous oversewing of these segments with a thin polypropylene suture (6.0) can be performed in order to adjust the lumen or prevent subsequent dilatation of the vascular segment [26]. Vein segments subject to postphlebitic changes should be excised and discarded [26].

**Fig. 7:**
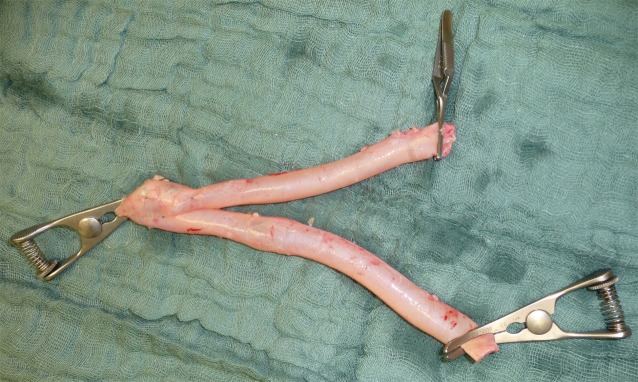
Creation of a new bifurcation using the superficial femoral vein. A new bifurcation for abdominal aortic repair is created using the valveless vein by means of an incision and a side-to-side suture (6.0 polypropylene)

**Fig. 8:**
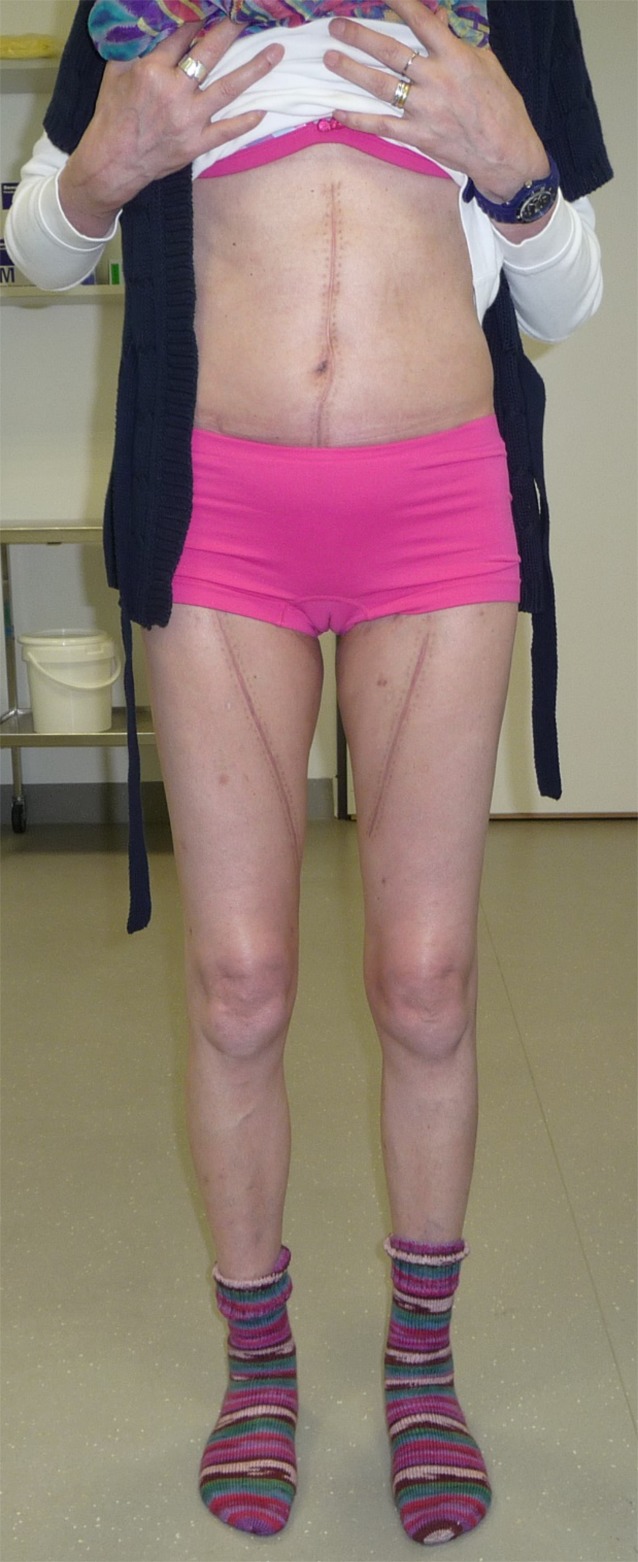
Postoperative clinical status at 3 months following bilateral harvest of the superficial femoral vein for mycotic abdominal aortic aneurysm. No visible edema in the lower legs under normal physical activity with no compression therapy

If necessary due to the extent of arterial reconstruction, the veins harvested from both legs, if they are of the required length, can be joined to form a long graft by means of an angled anastomosis or a neobirfurcation (Fig. 7 and 8) created for abdominal aortic repair by means of a side-to-side anastomosis. In the case of insufficient graft length, an alternative vein (e. g. an upper extremity vein) can be additionally integrated in the structure. The thus prepared venous graft can then either be placed in the carefully debrided and rinsed site following excision of the infected graft or native artery or, in the case of a different surgical indication, placed in the usual manner by means of tunnelling the bypass graft. Whenever graft tunnelling is required, the side branches should be oversewn and the use of vein clips dispensed with in order to avoid tearing off of the clips and the resulting risk of severe bleeding.

Once hemostasis has been achieved and several wound drains have been placed, wound closure following vein harvest is performed most simply with multilayer continuous suturing during fascial closure. It is important to ensure that a wound drain is also placed deep in the harvest bed. Depending on the amount of exudate, these drains can be left in place for several days. Postoperative mobilization of the patient is determined by the respective surgical indications and the individual course, particularly in the case of extensive abdominal procedures. If the arterial femoropopliteal axis is intact, an anti-embolism stocking can already be worn or the leg bound with an elastic bandage in the early postoperative phase.

## Harvest-related morbidity in superficial femoral vein use

We know from historical reports on the (no longer practiced) invasive treatment of deep vein thrombosis at the femoral level that SFV ligation to prevent pulmonary emboli caused clinically relevant edema in the operated leg in only a very small number of cases [52, 53]. In the case of a patent profunda femoralis vein, this was described in only 14 % of cases [54]; however, a high rate of venous congestion was reported if the common femoral vein was also affected [55]. Although deep venous reflux plays a lesser role in the formation of congestion-related venous ulceration compared with reflux in the superficial veins, it is more pronounced if clinical symptoms are more advanced [56–59].

From the outset of SFV use as a vascular replacement, fears understandably circulated regarding harvest-related impairment to venous return and possible associated acute and chronic venous complications, including the risk of amputation of the affected limb. In the comparative series on femoropopliteal reconstructions using the SFV or GSV published in 1987, Schulman et al. analyzed ankle circumference in the affected leg and observed a decline in circumference increase of 0.5–1 cm over time following SFV harvest. None of the 116 patients who underwent SFV bypass surgery were affected by long-term disability caused by edema or ulceration in the further course [1]. Coburn, with his own working group, was the second author to devote himself to the technique applied only by Schulman et al. up to that point and, after presenting the results, found himself confronted with an intensive discussion, as in contrast to Schulman et al. the publication reported serious complications following SFV harvesting [4]. In a small series of seven patients in 1993, Coburn et al. described two cases of severe venous outflow obstruction in the form of phlegmasia following SFV and popliteal vein harvest. Emergency venous bypass using a polytetrafluoroethylene (PTFE) graft was necessary in one case, while limb amputation above the knee was required in another case [4]; however, vein harvest was performed to far below the knee, in some cases to distal of the anterior tibial vein inflow, in five out of seven cases, in addition to which preoperative phlebographic assessment of the deep vein system had been dispensed with. They observed severe chronic edema that responded poorly to local compression therapy in three further cases involving extensive vein harvest of this kind. In all three of these cases, however, the vein was similarly harvested to the distal popliteal vein [4]. In contrast to this study Sladen et al. reported on a series of 25 interventions involving SFV harvest performed primarily for critical ischemia [26]. With the exception of one case, they avoided vein harvest to below the knee. Clinically relevant edema as a result of vein harvest was seen in four cases (20 % of cases at 1 year); however, one patient reported significantly more painful edema at 1 year. In this case again, extensive vein harvest to the level of the distal popliteal fossa had been performed [26].

Clagett’s working group concentrated intensive efforts on the clinical application of the SFV in a variety of indications and also analyzed the potential negative effects of harvesting on the residual venous system. By means of clinical examination, venous duplex sonography and venous functional tests, Wells et al. followed up 61 patients with 86 SFV harvests at 6‑month intervals [49]. No correlation was seen between postoperative swelling and the presence of a preoperatively intact GSV. Although plesmography indicated impaired venous outflow in 93 % following SFV harvest, this was reflected in a low reflux rate of only 11 % in lower leg veins. Moreover, whilst venous pressure during exercise was significantly elevated, this normalized rapidly and, in some cases, decreased again over time under serial observation [49]. No cases of venous ulceration or venous claudication were seen in this series. Instead, ultrasound revealed large caliber collateral vessels with a diameter of 4–6 mm from the popliteal vein in a central direction in 34 % of cases. Distinctly smaller caliber collateral vessels were identified in all other cases [49]. The same working group also investigated the effects of SFV harvest in terms of the development of acute postoperative venous hypertension and the need to relieve pressure by means of fasciotomy [60]. The authors analyzed data from 264 SFV harvests taken from 162 patients and found that the rate of fasciotomy in the aortoiliac axis (20.7 %) increased particularly in those cases with low preoperative ankle-brachial index (ABI) or higher intraoperative fluid administration. It was also more likely to be required in the case of concurrent harvest of the ipsilateral GSV. On the other hand, fasciotomy was not required when the SFV was used in other regions [60]. Thus, the authors recommended considering prophylactic fasciotomy in aortoiliac procedures in the case of severe ischemia and extensive vein harvesting or, at least, monitoring patients undergoing aortoiliac interventions correspondingly during the postoperative phase [60].

Using duplex sonography and venous function tests Modrall et al. clinically examined 27 legs following SFV harvest over a mean follow-up time of 70 months [61]. They found signs of chronic venous insufficiency with persistent swelling in 14.8 % of surgically treated legs (four cases). Edema was mild and easily controlled in two cases, one case of edema accompanied by skin changes was seen, as was one case of healed venous ulcer. Here again, a correlation was seen between the development of chronic venous insufficiency and concurrent harvest of the GSV and the SFV. An earlier case of GSV harvest on the other hand showed no negative effects in this respect. A further 46 patients could only be surveyed by telephone without undergoing clinical examination. Of these patients 15.2 % reported chronic edema in the leg operated on, whilst no respondents reported ulceration [61]. In the most recent study available, conducted in Mainz, Germany, the working group under Dorweiler reported mild edema in 21 % of cases at 24 months following SFV harvest to treat infection in the aortoiliofemoral region [12]. No severe impairment to venous outflow accompanied by marked clinical symptoms was seen in any of the 67 patients in this series (see Table 2).

**Table 2:** Long-term venous complications following superficial femoral vein harvest

Author	Year	*n*	Follow-up period	Edema	Measure	Severe venous complications Ulceration
Schulman et al. [1]	1987	65	–	Circumference increase of 0.5–1 cm compared with GSV group	*n. s.*	None
Coburn et al.[4]	1993	7	–	7	Compression stocking	2 (1 venous bypass, 1 amputation)
Sladen et al. [26]	1994	25	24 months	20 % (4 patients)	Lower leg compression stocking	1 patient with painful edema at 12 months no ulceration
Nevelsteen et al. [16]	1995	15	17 months	1/13	Compression stocking	None
Clagett et al. [10]	1997	41	32 months	10 % (4 patients)	Compression stocking	None
Wells et al. [49]	1999	86 legs	37 months	31 %	13 % compression stocking	None
Modrall et al.[61]	2007	27 legs	70 months	14.8 %	*n*. s.	1 resolved ulcer
Dorweiler et al.[12]	2014	84 legs	24 months	21 %	4 patients with compression stockings	None

*GSV* great saphenous vein, *n.s*. not significant

The role of thrombosis in the popliteal vein stump and tibial veins observed on postoperative duplex sonography following SFV harvest remains unclear. Although often clinically asymptomatic, this phenomenon was seen in up to 22 % of cases [12, 49]. Whilst embolic complications are not expected, one can speculate as to whether they promote chronic venous insufficiency. According to own experience, complete lysis of thrombosis occurs in 50 % of cases in the early postoperative months [12]; nevertheless, there is a fundamental risk of central thromboembolic complications following SFV harvest. Dhannisetty et al. reported on a series of 58 SFV harvests in 57 patients [62]. Of these procedures 47 % were performed due to vascular involvement in the context of cancer surgery, primarily for portomesenteric reconstruction. The authors observed a significantly higher incidence of thromboembolisms in cancer patients of 52 % compared with 10 % in cancer-free patients. All cases of venous thrombosis proximal to the SFV harvest site or in the contralateral extremity were seen in the cancer patients, as was the one case of pulmonary embolism. Venous thrombosis was not observed in patients receiving thromboprophylaxis for other indications (i.e. atrial fibrillation, hypercoagulability and history of thromboembolism) [62]. The authors concluded that normal postoperative thromboprophylaxis and duplex sonography of the deep vein system according to symptoms is adequate in patients without malignancies, whereas prolonged thromboprophylaxis in conjuction with routine duplex sonography of the deep vein system is indicated in cancer patients [62]. Other authors, in contrast, declare this approach to be inadequate and proposed routine full anticoagulation with low molecular weight heparin for 30 days; however, these authors’ experience was limited to dialysis shunt placement using the SFV [63].

Under the assumption that at least one intact valve in the popliteal vein, as well as a significant proximal collateral, is needed to guarantee unimpaired venous outflow, Santilli et al. carried out a pathoanatomical investigation of the deep vein system on cadaveric specimens to test this assumption [64]. According to this analysis, 15 cm of the popliteal vein in males (height 170 cm) and 12 cm in females (height 150 cm) can be harvested distal to the adductor hiatus in addition to the SFV in order to guarantee a “safe” harvest while preserving a valve-bearing popliteal segment and a venous collaterals without compromising venous outflow. This can be performed with 95 % confidence of preserving at least one valve and one collateral vein [64].

## Conclusion

The use of the SFV as an autologous vascular graft is a proven reconstructive procedure with a wide range of indications. The vein itself is ideal for aortoiliac repair, particularly in vascular surgery due to infections. Severe adverse sequelae in the residual deep venous system and the respective extremity are not anticipated if peripheral harvest is limited to the proximal popliteal vein.
